# Application of Metagenomic Next-Generation Sequencing in the Diagnosis of Pulmonary Infectious Pathogens From Bronchoalveolar Lavage Samples

**DOI:** 10.3389/fcimb.2021.541092

**Published:** 2021-03-11

**Authors:** Yuqian Chen, Wei Feng, Kai Ye, Li Guo, Han Xia, Yuanlin Guan, Limin Chai, Wenhua Shi, Cui Zhai, Jian Wang, Xin Yan, Qingting Wang, Qianqian Zhang, Cong Li, Pengtao Liu, Manxiang Li

**Affiliations:** ^1^ Department of Respiratory and Critical Care Medicine, The First Affiliated Hospital of Xi’an Jiaotong University, Xi’an, China; ^2^ Ministry of Education Key Lab for Intelligent, Networks and Networks Security, School of Electronics and Information Engineering, Xi’an Jiaotong University, Xi’an, China; ^3^ Department of Research and Development, Hugobiotech Co., Ltd., Beijing, China

**Keywords:** metagenomic next-generation sequencing (mNGS), bronchoalveolar lavage fluid (BALF), pulmonary infection, etiological culture, diagnostic

## Abstract

**Background:**

Metagenomic next-generation sequencing (mNGS) is a powerful method for pathogen detection. In this study, we assessed the value of mNGS for bronchoalveolar lavage (BAL) samples in the diagnosis of pulmonary infections.

**Methods:**

From February 2018 to April 2019, BAL samples were collected from 235 patients with suspected pulmonary infections. mNGS and microbial culture were performed to evaluate the effectiveness of mNGS in pulmonary infection diagnosis.

**Results:**

We employed mNGS to evaluate the alpha diversity, results suggesting that patients with confirmed pathogens had a lower microbial diversity index compared to that of patients with uncertain pathogens. For the patients admitted to the respiratory intensive care unit (RICU) or on a ventilator, they experienced a lower diversity index than that of the patients in the general ward or not on a ventilator. In addition, mNGS of BAL had a diagnostic sensitivity of 88.89% and a specificity of 14.86% in pulmonary infection, with 21.16% positive predictive value (PPV) and 83.87% negative predictive value (NPV). When rare pathogens were excluded, the sensitivity of mNGS decreased to 73.33%, and the specificity increased to 41.71%. For patients in the simple pulmonary infection group and the immunocompromised group, the main infection types were bacterial infection (58.33%) and mixed-infection (43.18%). Furthermore, mNGS had an advantage over culture in describing polymicrobial ecosystem, demonstrating the microbial distribution and the dominant strains of the respiratory tract in patients with different underlying diseases.

**Conclusions:**

The study indicated that mNGS of BAL samples could provide more accurate diagnostic information in pulmonary infections and demonstrate the changes of respiratory microbiome in different underlying diseases. This method might play an important role in the clinical use of antimicrobial agents in the future.

## Introduction

Pulmonary infections, caused by bacteria, viruses, or fungi, displaying an increasing incidence and mortality rate worldwide, are the third leading cause of total years of life lost (YLLs) (Naghavi et al., 2017). From the outbreaks of SARS in 2003 to COVID-19 in 2019, major infectious diseases caused by new pathogens have emerged endlessly. Timely etiological diagnosis is critical for proper clinical treatment. However, the clinical diagnosis remains difficult since the laboratory diagnosis has relied primarily on microbial culture, which is time-consuming and has relatively low sensitivities, especially in patients previously treated with anti-infective agents.

Nowadays, the era of precise public health and individualized medicine to treat pathogens had entered a new stage of metagenomic next-generation sequencing (mNGS) (Goodwin et al., 2016; Balloux et al., 2018; Leguia et al., 2019). The state-of-the-art mNGS technology is able to determine nucleic acid sequences more than 1 million base pairs in a single experiment (Forbes et al., 2017). The sequence reads generated can be linked to an accurate reference genome (or marker) database to identify pathogens (Forbes et al., 2017). It is unbiased and untargeted, so that it could enable wide-spectrum detection of known and unexpected pathogens or even unknown organisms. As a rapid microbiological diagnostic method for infectious diseases, mNGS had been recently introduced into clinical practice and provided vast quantities of etiological information, including species, strains, antibiotic resistance, and even virulence characteristics. Evidences had demonstrated the successful use of mNGS in clinical settings, including pneumonia, meningitis, liver abscess, endometritis with specimens from BAL, sputum, pleural fluid, and plasma (Langelier et al., 2018; Moreno et al., 2018; Miller et al., 2019).

However, when applying mNGS test into clinical microbiology laboratory diagnosis, numerous challenges remain in the interpretation of mNGS results (Goldberg et al., 2015; Gu et al., 2019). The utility of mNGS for BAL in pulmonary infections is yet to be explored. In this study, we evaluate the value of mNGS for BAL samples in the diagnosis of pulmonary infections and assess the pulmonary microbial communities in patients with different underlying diseases.

## Materials and Methods

### Patient Recruitment and Specimen Processing

Patients with suspected pulmonary infection were recruited from the First Affiliated Hospital of Xi ‘an Jiaotong University according to standard procedures. A total of 235 patients were investigated between February 2018 and April 2019. Specific diagnostic criteria of suspected pulmonary infection included new or deteriorated focal or diffuse infiltrating lesions on chest X-ray or computed tomography (CT), combined with at least one of the following four items: 1) fever >37°C, 2) cough, sputum production, hypoxia, or exacerbation of existing respiratory symptoms; 3) leukocytosis, 4) clinical signs of lung consolidation or moist rales. mNGS tests for the BAL samples were performed in patients who had suspected lung infection, paralleled with the conventional microbiological testing. Specimens from all patients were tested with the following traditional laboratory analysis: bacterial and fungal smear and culture, real-time polymerase chain reaction (PCR) for human betaherpesvirus 5 (cytomegaloviruses, CMV), human gammaherpesvirus 4 (Epstein-Barr virus, EBV), and *Mycobacterium tuberculosis*, serum antibodies for indirect immunofluorescence assay (IFA) for respiratory syncytial virus(RSV), influenza A/B virus, parainfluenza virus, adenovirus, *Legionella pneumophila*, *Mycoplasma pneumoniae*, and *Chlamydia pneumoniae*. Galactomannan antigen and (1/3)-β-D-glucans were also measured for fungi. Besides, Genexpert TB Test, enzyme-linked immunospot assay (T-SPOT) and tuberculin skin test were only preformed for patients with highly suspected tuberculosis. As a result, based on clinical manifestations, imaging findings, patient’s responsiveness to anti-infection therapy, together with the microbiological evidences, for 235 patients who were taken into this research, 220 patients were diagnosed with pulmonary infections. The remaining 15 patients were diagnosed with non-infectious diseases, including malignancies and non-infectious inflammatory diseases.

Based on the accompanying diseases, 220 pulmonary infection patients were classified into four groups: 1) the simple pulmonary infection group: patients without underlying diseases, 2) the chronic airway inflammation group: patients with chronic bronchitis, chronic obstructive pulmonary disease (COPD), or asthma, 3) the immunocompromised host pneumonia (ICH) group: patients diagnosed with autoimmune disease, post-splenectomy, and/or with long-term treatment with glucocorticoids, immunosuppressive agents, cytotoxic drugs, hematological malignancies, chemotherapy in the last 6 months, or solid organ transplantation; 4) other group: the remaining patients with other comorbidities except for chronic bronchitis, COPD, asthma or immunocompromised diseases. Patients with COPD, chronic bronchitis, or asthma and on immunosuppressants were classified in the ICH group.

### DNA Extraction and Sequencing

The collected bronchoalveolar lavage samples were sent to the clinical microbiology lab and histopathology lab within 2 h after the collection. Meanwhile, the remaining specimens were sent to MicrobeCode Biotech Co. Ltd (Xi’an, China) for sequencing and bioinformatics analysis. DNA was purified using TIANGEN DP316 kit. The sequencing library was prepared using NEBNext Ultra II DNA library Prep Kit according to manufacturer’s instruction. The library quantity and quality were assessed using Qubit and agarose gel electrophoresis. The purified library was then diluted, mixed, denatured, re-diluted, spiked with PhiX (equal to 1% of final DNA amount) following the MiniSeq System Denature and Dilute Libraries Guide, and then applied to an Illumina MiniSeq system for sequencing with the High Output Reagent Kit 150 cycles as described in MiniSeq Local Run Manager Software Guide.

### Bioinformation Pipeline

In order to generate high-quality data, the raw data generated by Miniseq were filtered to remove adapter, low-quality, low-complexity, and short reads (smaller than 35 bp), using an in-house program. Then the human sequences were excluded by mapping reads to the human reference genome (hg19) with the application of the Burrows-Wheeler Alignment, using SNAP software (Li and Durbin, 2009). The remaining data were aligned to a microbial genome database (NCBI; ftp://ftp.ncbi.nlm.nih.gov/genomes). The reference database used for this study contained 11,910 bacteria, 7,103 viruses, 1,046 fungi, and 305 parasites. Unique reads were defined as reads with global alignment, with the optimal identity higher than 95% to the reference sequence, and with the suboptimal identity lower than 90%. Finally, the microbial compositions of the samples were obtained. The statement of deposition for sequence reads had been submitted to NCBI under the project accession number PRJNA-644754.

### Criteria for a Positive mNGS Result

Infectious pathogens were defined as the microorganisms with pulmonary pathogenicity reported in the literature. The infectious bacteria (including *Mycobacterium tuberculosis*), viruses, or fungi were considered positive if they met any of the following thresholds of mNGS: 1) >30% relative abundance at the genus level in bacteria, viruses, or fungi; 2) histopathological examination and/or culture positive with at least 50 unique reads of a single species of bacteria or fungi; 3) for *Mycobacterium tuberculosis*, at least one unique read, due to the difficulty of DNA extraction and low possibility for contamination (Langelier et al., 2018). Study suggested that even though only one unique read was identified by mNGS, clinical nucleic acid test results of *Mycobacterium tuberculosis* were positive (Li et al., 2018). Microbes identified by mNGS were classified as confirmed pathogens if also positive for any of the clinical testings (including pathology, culture, smear, antigen or antibody detection, PCR, etc.). Microbes were considered potential pathogens if they were identified as positive infectious pathogen by only mNGS but not by any other clinical tests. All other microbes that were identified by mNGS but did not meet the criteria for a positive mNGS result, were considered uncertain pathogens.

### Calculation of Alpha Diversity

A Microbial diversity index is used to describe the community species diversity. The Shannon’s Diversity Index was utilized to assess the alpha diversity of all microbial presented in BAL fluids from 235 patients and performed using vegan package of R software (https://www.r-project.org). The formula was defined as $-\sum_{i=1}^{k}p_{i}\log(p_{i})$ (Finotello et al., 2018), where k was the total number of species, and *p_i_* was the proportional abundance of species i. **Supplementary Table 1** addressed the detail values for each patient.

### Statistical Analysis

Following the extracted data, 2 × 2 contingency tables were derived to determine the sensitivity and McNemar test for discrete variables when appropriate. Data analyses were performed using SPSS18 and Graphed Prism7 software. P values smaller than 0.05 were considered statistically significant, and all tests were two-tailed.

## Results

### Patient Characteristics and mNGS Results

Demographic features of recruited patients in the current study were presented in **Table 1**. Within the 235 patients included, 155 were males and 80 were females. The mean age was 54.7 years (14–94 years). Among them, 74.47% (175/235) patients had underlying diseases, including cardiovascular diseases (42 cases), cerebrovascular diseases (10 cases), diabetes (13 cases), chronic airway inflammation (13 cases), organ transplantation (12 cases), hematologic neoplasm (3 cases), autoimmunity diseases (3 cases), chronic kidney diseases (10 cases), post-splenectomy (1 case), malignant tumor (24 cases), post-pneumonectomy (2 cases), and other complications (66 cases). The comparisons of mNGS results, conventional microbiology methods (smear, culture, and other etiological examination), and histopathological results were shown in **Supplementary Table 1**. A total of 89.78% (211/235) patients with suspected pulmonary infections received empirical antibiotics, and 92.27% (203/220) patients with final diagnosis of pulmonary infection received empirical antibiotics.

**Table 1:** Characteristics of patients.

Characteristics	Value
Number	235
Patient age (median, range)	54.7, 14–94
Sex (% male)	65.96%
Intensive care unit	61.97%
Ventilator	18.72%
Complication	175
Cardiovascular Disease	42
Cerebrovascular disease	10
Diabetes	13
Chronic airway inflammation	31
Chronic bronchitis	3
Chronic obstructive pulmonary disease	23
Asthma	9
Organ transplantation	12
Hematologic neoplasm	3
Autoimmunity disease	23
Chronic kidney diseases	10
Non-steroids and immunosuppressive drugs	3
Steroids or immunosuppressive drugs	7
Post-splenectomy	1
Malignant tumors	24
Newly diagnosed	19
Received chemotherapy or radiation	3
Post-pneumonectomy	2
Others	66

### Microbial Diversity and Pathogen Detection

**Figure 1:**
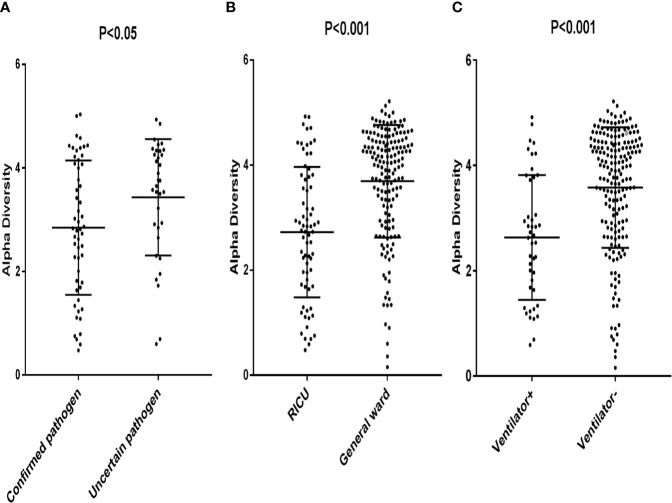
Each data point represented a patient whose Alpha Diversity Index was plotted on the y-axis. **(A)** Subjects were grouped according to confirmed pathogen *vs.* uncertain pathogen; **(B)** Subjects were grouped according to pathogens from patients in RICU *vs.* pathogens from patients in general ward. **(C)** Subjects were grouped according to pathogens from patients on a ventilator *vs.* pathogens from patients not on a ventilator.

The patients with confirmed pathogens had a significantly lower alpha diversity (Median, 2.85; interquartile range [IQR], 1.80–4.08) compared to patients with uncertain pathogens (Median, 3.43; IQR, 2.86–4.31) (p < 0.05) (**Figure 1A**). Patients in RICU also had a lower diversity compared to patients in general ward (Median, 2.73; [IQR, 1.77–3.75] *vs.* 3.69 [IQR, 3.08–4.46] (p < 0.05) (**Figure 1B**). Moreover, the microbial diversity was significantly decreased in patients on a ventilator compared to those not on a ventilator (Median, 2.63; [IQR, 1.67–3.74] *vs* 3.57 [IQR, 2.88–4.34] (p < 0.05) (**Figure 1C**). These results indicated that the alpha diversity could be an indicator for an active infection and the level of severity for the infection.

### Comparison and Concordance of mNGS and Culture Method

Of the 220 patients with final diagnosis of pulmonary infections, the comparison between mNGS and culture method indicated that mNGS had a diagnostic sensitivity of 88.89% (95% CI: 75.15–95.84%) and a specificity of 14.86% (95% CI: 10.11–21.20%). The positive predictive value (PPV) (21.16%, 95% CI: 15.71–27.82%) was significantly lower than the negative predictive value (NPV) (83.87%, 95% CI: 65.53–93.91%). When excluding rare pathogens which were not interpreted as pathogenic microbes, the sensitivity of mNGS decreased to 73.33% and the specificity increased to 41.71% (**Table 2**).

**Table 2:** Performance of metagenomic next generation sequencing (mNGS) compared with culture test in pulmonary infection.

	Sensitivity (95% CI)	Specificity (95% CI)	PPV (95% CI)	NPV (95% CI)
mNGS^a^	88.89% (75.15–95.84%)	14.86% (10.11–21.20%)	21.16% (15.71–27.82%)	83.87% (65.53–93.91%)
mNGS^b^	73.33% (57.79–84.90%)	41.71% (34.39–49.41%)	24.44% (17.64–32.73%)	85.88% (76.25–92.18%)

a According to the mNGS positive standard.

b Excluded rare pathogens which was not interpreted as pathogenic microbes.

**Figure 2:**
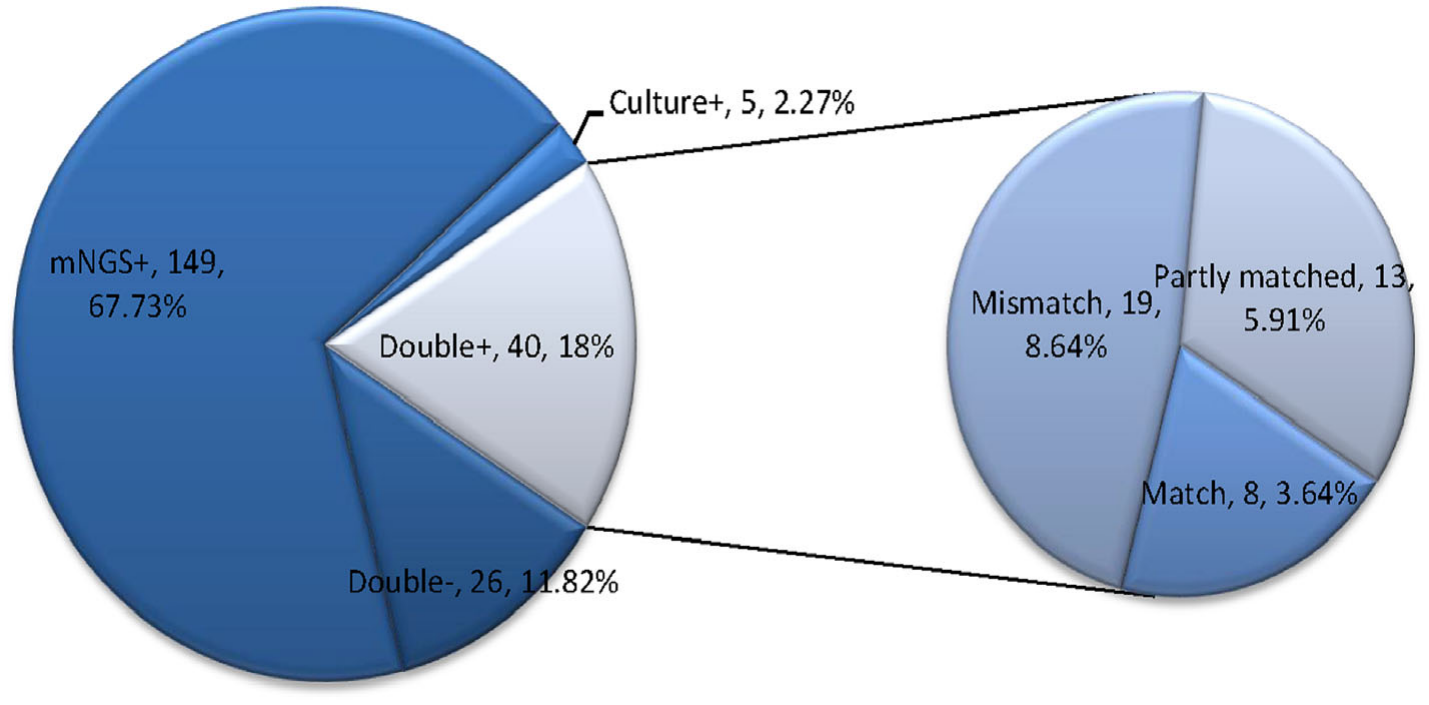
This figure showed the positivity distribution of mNGS and culture in 220 patients with a final diagnosis of pulmonary infection. Among the 220 patients, the number of patients with only positive mNGS results was 149, and five samples were only culture-positive. For the double-positive group, 52.5% (21/40) of them showed completely or partly matched results. Also, in this group, there were 19 cases showing conflicts between the results of mNGS and culture.

Moreover, among the 220 infectious patients, the mNGS method indicated concordant results compared to culture test in 66 samples (40 double-positive and 26 double-negative) with a matching accuracy of 30% (66/220). And mNGS identified more infectious pathogens than culture test. A total of 149 cases (67.73%) were positive for pathogens detected by mNGS only, and five cases (2.27%) were positive by culture test only. Among the 40 double-positive samples (6 in the simple pulmonary infection group, 4 in the chronic airway inflammation group, 13 in the ICH group, and 17 in other group), more than half (21/40) of them showed completely (8 cases) or partly (13 cases) matched results between mNGS and culture. In contrast, the remaining 19 cases were totally mismatched (**Figure 2**).

### The Detailed Information of Samples With Conflicting Results

In 40-double positive patients, there were 19 cases shown a paradoxical result between mNGS and culture test. It is worth mentioning that, within the 19 discrepancy cases, there was one case, in which culture results were concordant with mNGS but did not meet the mNGS positive criteria. For the remaining 18 cases, culture results were completely different from mNGS results. Within all the 19 mNGS positive results, viruses were detected in eight cases by mNGS, with only one case confirmed by real-time PCR (P96). Fungal infections detected in seven cases by mNGS were undetectable by culture. Among the seven cases, P8, P17, P51, P96 identified *P. jirovecii*, P99 identified *Aspergillus niger*, P112 identified *Candida albicans*, P217 identified *Candida tropicalis* and *Candida glabrata. P. jirovecii* requires a special culture medium and cannot be routinely cultured. Culture from respiratory specimens for *Aspergillus niger* requires a long culture period. Besides, bacteria detected in eight patients by mNGS were not interpreted as the pathogen. In P98, the *Mycobacterium tuberculosis* identified by mNGS was confirmed by acid-fast bacteria identification of smearing. The remaining bacteria (*Mycobacterium triviale*, *K. pneumoniae*, *P. aeruginosa*, *Staphylococcus haemolyticus*) detected by mNGS in nine patients were considered as the latent infection (**Supplemental Table 2**). Moreover, pathogen isolation might not be possible, especially when antibiotic treatment has already been initiated.

### Infection Types in Different Underlying Disease

**Figure 3:**
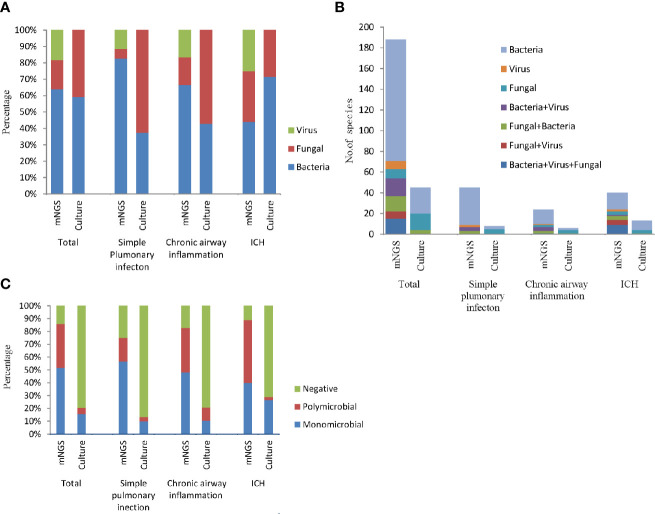
The bar chart represented the infection types in patients with different underlying diseases according to the positive results of culture and mNGS. **(A)** Fraction of species (bacteria, fungi, and viruses) in different groups; **(B)** Number of infection type; **(C)** Proportion of polymicrobial, monomicrobial infection, and negative cases of pulmonary infection detected using mNGS and culture method.

According to the mNGS results, the most frequently detected pathogens were bacteria, followed by the viruses and fungi (**Figure 3A**). More than half of all infected patients were diagnosed with simple bacterial infection (53.18%, 117/220). Moreover, in the simple pulmonary infection group (60%, 36/60) and the chronic airway inflammation group (48.28%, 14/29), the type of the infection was also dominated by simple bacterial infection. However, the main infection type in the immunocompromised host group was mixed infection (42.22%, 19/45, nine cases of bacteria-fungi-virus co-infection, five cases of fungi-virus co-infection, one case of bacteria-virus co-infection, four cases of bacteria-fungi co-infection) (**Figure 3B**). Notably, viruses (82.98%, 39/47) and fungi (80.43%, 37/46) detected by mNGS were commonly seen in the mixed infection cases, while bacteria could only be detected in 29.09% (48/165) of the mixed infection cases (**Figure 3A**). Contrastively, Microbial culture only identified 45 positive results in 220 patients. The most common infection type in the total participants and the ICH group were also the bacterial infection (**Figure 3B**). The feasibility of the culture method in detecting fungi and bacteria was similar (10 and 13.18%, respectively) (**Figure 3A**). No virus culture was performed in this study.

In addition, mNGS diagnosed more polymicrobial infection (two or more microorganims) than culture method (34.09 *vs.* 5%). According to the mNGS results, polymicrobial infection appeared more frequently in the chronic airway inflammation group (34.48%) and the immunocompromised host group (48.89%) compared to the simple pulmonary infection group (18.33%). While as the results of culture assay suggested, polymicrobial infections were most likely to be seen in the chronic airway inflammation group (**Figure 3C**).

### Distribution of Microbiome in Different Groups

**Figure 4:**
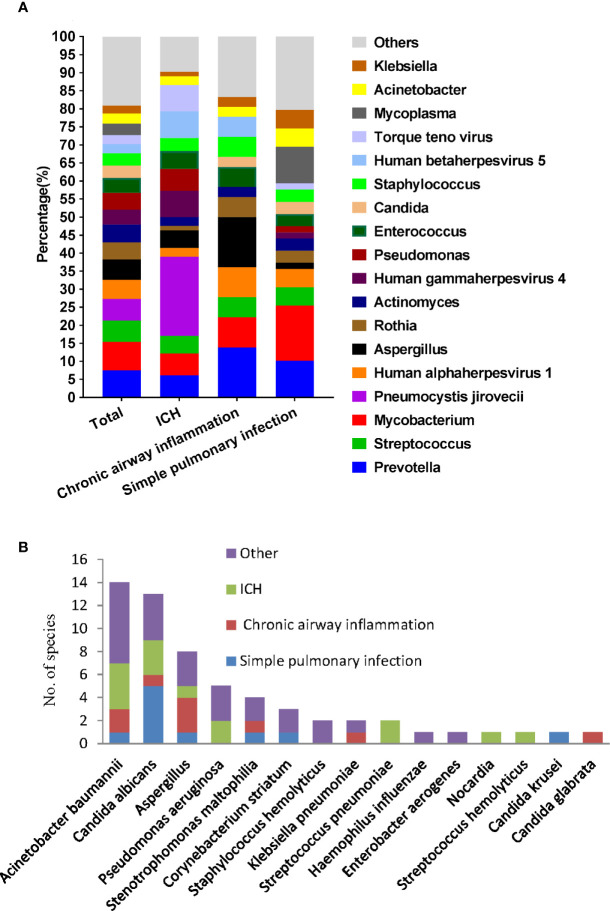
Microbiological profile of the 220 infection patients assessed by mNGS and culture. **(A)** Proportion at the genus level of the 15 most abundant bacterial genera identified by mNGS. **(B)** Number of pathogens identified by culture method.

We then used mNGS and culture method to explore the microbiome of patients with different underlying diseases and immune backgrounds. Among the 220 patients, the most frequent genus identified by mNGS was *Prevotella*, followed by *Mycobacterium* and *Streptococcus*. While *Mycobacterium*, *Prevotella*, and *Mycoplasma* were more frequent in simple pulmonary infection group. *Prevotella*, *Aspergillus*, and Human alphaherpesvirus 1 resulted in a higher proportion in the chronic airway inflammation group. It is worth mentioning that, *P. jirovecii*, Human gammaherpesvirus 4, Human betaherpesvirus 5, Torque teno virus exhibited a higher percentage in the immunocompromised host group (**Figure 4A**). Notably, among 10 kidney transplant patients, mNGS detected *P. jirovecii* in nine cases.

The causative microbe in 45 culture-positive patients were shown in **Figure 4B**. In the simple pulmonary infection group, the most detected causative microbe was *Candida albicans.* In contrast, *Acinetobacter baumanni* and *Aspergillus* were the most frequent microbes in the ICH and the chronic airway inflammation group, respectively.

## Discussion

In this study, we compared mNGS and culture method for BAL samples systematically, with results suggesting the advantages of the mNGS in several aspects. Our research indicated that the species diversity index could serve as an indicator of active infection and the severity of infection. Furthermore, mNGS performed well in the pathogen detection and polymicrobial infection diagnosis. It could also identify more respiratory microbiome and dominant pathogens in patients with different underlying diseases.

The state-of-the-art technology mNGS is well-known to have the advantage of pathogen detection by direct sequencing of the extracted DNA from specimens, having a revolutionary impact on microbiological diagnosis. mNGS provides a wide range of microbial profiles independent of the priori selection of targeted pathogens and is able to detect many potential infectious agents in a single assay (Goldberg et al., 2015). Our study showed that mNGS had a diagnostic sensitivity of 88.89% and NPV of 83.37% in identification of all pathogens. However, several rare or opportunistic pathogen genera (including *Enterococcus*, *Veillonella*, *Rothia*, *Prevotella*, etc.) may challenge the interpretation of the mNGS results. When excluding rare pathogens which were not interpreted as the pathogenic microbes, the sensitivity of mNGS decreased to 73.33%, and the NPV increased to 85.88%. Although mNGS is a far more sensitive technology than traditional pathogen culture, our specificity and PPV positive rate of mNGS appeared to be lower than expected. One possible reason was the false-negative results in culture assays caused by fastidious microorganisms and by the previous initiated antibiotics treatment before the collection of BAL samples (Scott et al., 2000; Rhodes et al., 2010; Harris et al., 2017). Compared to culture test, the mNGS was less likely to be affected by the prior antibiotic usage (Rhodes et al., 2010; Miao et al., 2018). Another reason may be that BAL derived from the respiratory tract was typically mixed with oral flora and colonizers, contributing to the relatively higher false-positive results of mNGS compared with other sample types. This study also provided evidences for the consistency between mNGS and culture test in 66 specimens (40 double-positive and 26 double negative). Among the 40 double-positive samples, 52.5% of patients presented completely or partly matched results. The poor overall matching rate in the pie chart of **Figure 2** between mNGS and culture may attribute to the low positive rate and narrow detection range of etiological culture. Moreover, complications exist in determining whether a microbe is dead or alive (Emerson et al., 2017). mNGS can detect free DNA floating in the samples, making it possible to identify DNA of non-viable bacteria which had been there but not currently present. Based on the number of reads and relative abundance, mNGS could be used for semi-quantitative detection (Hu et al., 2016; Ai et al., 2018), which may potentially benefit the differentiation of the live microbes from fragmented DNA sequences.

Evidences have shown that lungs are accompanied with the shift in microbial composition and diversity during diseases (Willner et al., 2009; Hilty et al., 2010; Erb-Downward et al., 2011; Cho and Blaser, 2012). The exacerbations of pulmonary diseases are occasions of respiratory tract microbial ecosystem dysbiosis (Dickson et al., 2014). Our study indicated that the patients with confirmed pathogens had a lower alpha diversity compared with the patients with uncertain pathogens. Besides, patients admitted in the RICU or on a ventilator had a lower diversity compared with patients in the general ward or not on a ventilator, consistent with other studies (Cox et al., 2010; Zhao et al., 2012; Borghi et al., 2017; Langelier et al., 2018; Pragman et al., 2019). The previous study had demonstrated that community diversity would decrease in patients with confirmed pathogen (Langelier et al., 2018). More evidences also suggested that the decreased microbial diversity was positively correlated with the level of severity for disease in the gut and lung (Cox et al., 2010; Zhao et al., 2012; Borghi et al., 2017; Pragman et al., 2019). Our results revealed that the species diversity index could serve as an indicator of active infection and the severity of the infection.

In our study, mNGS not only identified *Mycobacterium tuberculosis* as the most common pathogen in the total patients but also in the simple pulmonary infection group. Similar phenomena were observed by (Huang et al., 2020) and (Miao et al., 2018). In addition, *P. jirovecii*, Human gammaherpesvirus 4, Human betaherpesvirus 5, and Torque teno virus had shown higher percentages in the immunocompromised host group. Torque teno virus, a small, non-enveloped single-stranded DNA virus that causes chronic, possibly lifelong viremias in most people, is not considered a common cause of pulmonary infection but increasingly recognized as a potential pathogen in immunocompromised patients (Maggi et al., 2011; Wootton et al., 2011). Thus, interpretation of pathogens in immunocompromised patients should be taken with serious consideration and guided by clinical manifestations and relevant laboratory examination results. In comparison, the most frequent microbes detected by culture method were *Acinetobacter baumannii*, *Candida albicans*, and *Aspergillus.* The recent publication by Pan et al. also recognized that *P. jirovecii* and CMV were the most frequent pathogen revealed by mNGS in 13 ICH patients (Pan et al., 2019). Besides, *Acinetobacter baumanni* was the most frequent pathogen detected by culture method (Pan et al., 2019). However, this research might have an enrolled bias due to the fact that the patients admitted to our hospital were those who might had already experienced the difficulty in the diagnosis or had poor therapeutic response to the conventional anti-infective agents in external hospitals.

In the present study, patients who had underlying diseases such as chronic airway inflammation and immune damage, might have an increased risk for viral, fungal, and even mixed infections. Most of the fungi were non-pathogenic in healthy individuals, however, patients with underlying diseases such as cancer or other immunocompromised conditions were at higher risk for fungal infections (Romani, 2004). Furthermore, viruses and fungi detected by mNGS were always in mixed infection cases. Literature indicated that the presence of one microorganism generated a niche for other pathogens to colonize or suppressed the colonization of other micro-organisms (Brogden et al., 2005). Our study indicated that infection with viruses and fungi might predispose the host to colonization by other microorganisms. Recently, the significance of polymicrobial infections had also been recognized. This study suggested that mNGS had an advantage over culture in describing polymicrobial ecosystems. And polymicrobial infections were more frequently seen in patients with underlying diseases. However, culture methods identified few polymicrobial infections in the ICH group, possibly because pathogens in immunocompromised patients are fastidious to grow or non-cultivable (Fenollar and Raoult, 2007). Thus it is difficult to diagnose the co-occurrence of opportunistic infections in immunocompromised patients by culture methods (Pan et al., 2019).

The application of mNGS pathogens detection was commonly seen in the normally sterile specimens, such as cerebrospinal fluid (CSF) and brain biopsies, to simplify the clinic detection of microorganisms (Wilson et al., 2014; Alonso et al., 2018; Wilson et al., 2019). Recently, mNGS not only detected infection pathogens, but also reflected normal microbiota, transient colonizers, and sample contamination, especially in respiratory specimens. Also, several studies advised that the lungs were not sterile (Charlson et al., 2011; Bassis et al., 2015; Dickson and Huffnagle, 2015), and the sampling process could not guarantee sterility, which might lead to numerous challenges for the reasonable explanation of mNGS for broad-spectrum pathogen detection in the clinical laboratory. Despite of this pitfall, mNGS had been applying in the diagnosis of infectious diseases successfully. In the future study, we will continue to optimize the mNGS criteria for pathogen identification for more reliable results.

There were four limitations in this study. Firstly, pathogens detected by mNGS was not validated with an additional molecular method at genetic level. Secondly, respiratory microbiome data were not obtained from healthy population as a baseline microbial community, therefore it was hard to characterize the lung microbiome of patients and to generate an optimal threshold for pathogen identification. Thirdly, because of the constraints of time and laboratory conditions, we only performed mNGS for DNA to test for bacteria, fungi, and DNA viruses without RNA virus detection and further virus culture. Finally, due to the small sample size of our study, it would be difficult to define a cutoff value of the diversity index that could distinguish different underlying disease groups. The clinical value of diversity index still needs further studies. Meanwhile, the accurate interpretation of mNGS and the application of diversity index should be combined with clinical manifestations and conventional laboratory-based diagnosis.

## Data Availability Statement

The datasets generated for this study can be found in the NCBI, BioProject: PRJNA644754, https://www.ebi.ac.uk/ena/browser/.

## Ethics Statement

The studies involving human participants were reviewed and approved by Xi’an Jiaotong University School of Medicine First Affiliated Hospital Ethics Committee. Written informed consent to participate in this study was provided by the participants’ legal guardian/next of kin.

## Author Contributions

YC: designing the experiment, collecting data, analyzing data, writing the manuscript. WF, LC, CZ, XY, WS, JW, QW, QZ, CL, PL, ML: designing the study protocol, collecting data, writing the manuscript, submitting to the publication. KY, LG, HX, and YG: technical support, implementing data acquisition, and revising the manuscript. All authors contributed to the article and approved the submitted version.

## Conflict of Interest

Authors HX and YG were employed by Hugobiotech Co., Ltd., Beijing, China.

The remaining authors declare that the research was conducted in the absence of any commercial or financial relationships that could be construed as a potential conflict of interest.

## Abbreviations

RICU, respiratory intensive care unit; NSCLC, non-small cell lung cancer; SCLC, small cell lung cancer; YLLs, Years of life lost; IQR, interquartile range.
