# Supplementary material for: Application of Metagenomic Next-Generation Sequencing in the Diagnosis of Pulmonary Infectious Pathogens From Bronchoalveolar Lavage Samples
Source: Front Cell Infect Microbiol. 2021 Mar 11;11:541092. doi: 10.3389/fcimb.2021.541092 (PMC7991794; doi:10.3389/fcimb.2021.541092)
Supplement: Supplementary file 1 [file Table_1.docx]

Supplemental Table S1: Clinical and Microbiologic Data

| ID | mNGS pathogen | Classical Microbiology | Pathology results | Underlying disease | Pulmonary disorders |
| --- | --- | --- | --- | --- | --- |
| 1 | Human alphaherpesvirus 1^a^ | FluB (IgM) | Chronic mucositis | Others | Pulmonary infection |
| 2 | *Aspergillus fumigatus* | FluB (IgM) | Non | Urinary infection | Pulmonary infection |
| 3 | Human alphaherpesvirus 1^a^ | Non | Lung tissue | Hypertension | Pulmonary infection |
| 4 | *Mycoplasma felis* | Non | Adenoma-like hyperplasia | Non | Pulmonary infection  Right upper lobe adenomatous hyperplasia |
| 5 | *Rothia mucilaginosa^a^* | Non | Chronic mucositis | Non | Pulmonary infection  COPD |
| 6 | *Mycobacterium* *tuberculosis* | *Tuberculosis* | Chronic mucositis | Non | Tuberculosis  COPD |
| 7 | *Streptococcus pneumaoniae* | Non | Non | Hypertension  Septic shock | Pulmonary infection |
| 8 | Torque teno virus  *Pneumocystis jirovecii* | Candida albicans  CMV  EBV | Non | Kidney transplantation  Coronary heart disease | ICH |
| 9 | *Streptococcus genus*  Torque teno virus  *Pneumocystis jirovecii* | Fungus  CMV | Chronic mucositis | Kidney transplantation | ICH |
| 10 | *Rothia mucilaginosa^a^* | *Stenotrophomonas maltophilia* | Chronic mucositis | Non | Pulmonary infection  Pleural effusion |
| 11 | Non | FluB | Chronic mucosal inflammation and a little inflammatory exudate | Non | Pulmonary infection |
| 12 | *Streptococcus pneumoniae* | Non | Chronic mucositis | Uremia (Hematodialysis） | Uremic pleurisy?  Tuberculous pleuritis? |
| 13 | *Actinomyces oris^a^* | *Candida albicans* | Chronic mucositis | Non | Fungal infection |
| 14 | Non | Non | Chronic mucositis | Hypertension | Pulmonary infection  Asthma |
| 15 | *Staphylococcus haemolyticus**^a^* | *Tuberculosis*  Fungus | Granulomatous inflammation of the lung tissue, considering tuberculosis | Non | Tuberculosis |
| 16 | Human alphaherpesvirus 1^a^ | Non | Chronic mucositis | Mixed connective- tissuedisease  Coronary heart disease | ICH |
| 17 | *Actinomyces odontolyticus^a^*  Human gammaherpesvirus 4  Human betaherpesvirus 5  *Pneumocystis jirovecii* | *Acinetobacter baumannii*  EBV  Fungus | Non | - IgA nephropathy | ICH |
| 18 | Non | Non | - Lung tissue | Non | Pulmonary infection |
| 19 | *Achromobacter xylosoxidans*  Human betaherpesvirus 5  *Aspergillus fumigatus* | FluA (IgM) | Non | Coronary heart disease  Hypertension | Pulmonary infection  COPD |
| 20 | *Prevotella melaninogenica^a^*  Merkel cell polyomavirus^a^  Human betaherpesvirus 7 | Non | Lung tissue | Muitinodular goiter | Pulmonary infection |
| 21 | *Mycobacterium tuberculosis* | *Tuberculosis* | Chronic mucositis | Non | Tuberculosis |
| 22 | Torque teno virus 1  Human alphaherpesvirus 1^a^  Human betaherpesvirus 7  *Pneumocystis jirovecii* | CMV  Fungus | Non | Kidney transplantation  Hypertension | ICH |
| 23 | *Actinomyces graevenitzii^a^*  Torque teno virus 6  *Pneumocystis jirovecii* | *Acinetobacter baumannii*  Fungus | Non | Hematologic neoplasm | ICH |
| 24 | *Rothia mucilaginosa^a^* | Non | Chronic mucositis | Coronary heart disease | Pulmonary infection  Pulmonary fibrosis |
| 25 | Non | *Human staphylococcus* | Non | Non | Bronchiectasis and infection  Lung abscess  Pyothorax |
| 26 | *Moraxella catarrhalis* | Non | Chronic mucositis | Patent foramen ovale | Pulmonary infection  COPD |
| 27 | Non | Non | Non | Non | Pulmonary infection |
| 28 | *Acinetobacter baumannii*  *Pneumocystis jirovecii* | *Acinetobacter baumanii*  CMV | Non | Eosinophilic granulomatous vasculitis  Post-pneumonectomy | ICH |
| 29 | Non | *Acinetobacter baumanii*  EBV | Non | Atrial fibrillation | Pulmonary infection  COPD |
| 30 | *Enterococcus faecium^a^* | *Aspergilus flavus* | Non | Cerebral infarction | Pulmonary infection |
| 31 | Human gammaherpesvirus 4  *Candida albicans* | Non | Poorly differentiated squamous cell carcinoma with necrosis and cavity formation, pneumonia and focal abscess | Non | Pulmonary infection  NSCLC (Newly diagnosed) |
| 32 | Non | Non | Chronic mucositis | Non | Pulmonary infection |
| 33 | Non | Non | Chronic mucositis | Non | Pulmonary infection |
| 34 | *Rothia dentocariosa^a^*  *Aspergillus fumigatus* | *Aspergillus fumigatus* | Non | Fatty liver disease | Pulmonary infection |
| 35 | *Trueperella pyogenes^a^* | Non | Chronic mucositis | Diabetes | Pulmonary infection  Pleural effusion |
| 36 | Human mastadenovirus B | Non | Chronic mucositis | Non | Pulmonary infection |
| 37 | *Prevotella sp. C561^a^* | Non | Organizing pneumonia | Non | Pulmonary infection |
| 38 | Human betaherpesvirus 7 | Non | Chronic mucositis | Peritonitis | Pulmonary infection |
| 39 | Non | Non | Interstitial fibrous tissue hyperplasia | Non | Pulmonary infection |
| 40 | *Mycobacterium tuberculosis* | *Tuberculosis* | Lung tissue | Non | Tuberculosis |
| 41 | *Prevotella intermedia^a^* | Non | Chronic mucositis | - Postsplenectomy | ICH |
| 42 | *Prevotella melaninogenica^a^* | Fungus | Chronic mucositis | Non | Pulmonary infection  Pulmonary fibrosis |
| 43 | *Mycoplasma pneumoniae* | Non | Chronic mucositis | Non | Pulmonary infection |
| 44 | *Rothia mucilaginosa^a^*  *Mycobacterium tuberculosis*  Human gammaherpesvirus 4 | EBV | Non | Chronic hepatic injury | Pulmonary infection |
| 45 | *Nocardia brevicatena* | Fungus | Chronic mucositis | Hypertension | Pulmonary infection |
| 46 | *Haemophilus parainfluenzae*  *Aspergillus niger* | Non | Chronic mucositis | Non | Pulmonary infection |
| 47 | *Mycobacterium triviale*  Human betaherpesvirus 6A | *Corynebacterium striatum Candida* | Non | - Duret hemorrhage   Hypertension | Pulmonary infection |
| 48 | Human alphaherpesvirus 1  Human gammaherpesvirus 4 | EBV | Non | - ANCA-associated vasculitis   Coronary heart disease  Hypertension | ICH |
| 49 | Human betaherpesirus 5  *Pneumocystis jirovecii*  *Aspergillus fumigatus* | CMV  Fungus | Non | Kidney transplantation | ICH |
| 50 | *Rothia mucilaginosa^a^* | *Acinetobacter baumannii* | Non | - Hypoproteinemia | Pulmonary infection |
| 51 | *Pneumocystis jirovecii* | *Acinetobacter baumannii* | Non | Anaphylatic purpura nephritis | ICH |
| 52 | Non | Non | Chronic mucositis | Uremia | Pulmonary infection |
| 53 | *Veillonella parvula^a^* | Non | Well-differentiated squamous cell carcinoma | Non | NSCLC (Newly diagnosed) |
| 54 | *Rothia mucilaginosa^a^*  *Cellulomonas flavigena^a^* | Non | Chronic mucositis | Non | Pulmonary infection  COPD |
| 55 | *Klebsiella pneumoniae* | Non | Non | Non | ICH (NSCLC after chemotherapy) |
| 56 | *Rothia mucilaginosa^a^*  *Candida albicans* | Non | Chronic mucositis | Non | Pulmonary infection |
| 57 | *Mycobacterium tuberculosis* | Non | Granulomatous inflammation of the lung tissue, PAS +, suggesting cryptococcal pneumonia | Non | Pulmonary infection |
| 58 | Non | *Tuberculosis* | Chronic mucositis | Non | Tuberculosis |
| 59 | Non | Non | Lung tissue | Others | Pulmonary infection |
| 60 | *Rothia mucilaginosa^a^ Pneumocystis jirovecii* | Non | Non | kidney transplantation | ICH |
| 61 | *Prevotella melaninogenica^a^* | FluB(IgM) | Non | Cholecystectomy | Pulmonary infection |
| 62 | *Prevotella intermedia^a^* | *Tuberculosis* EBV(IgM)  *Mycoplasma pneumoniae* (IgM) | Chronic mucositis | Systemic lupus erythematosus | ICH  Tuberculosis |
| 63 | *Mycoplasma pneumoniae* | *Mycoplasma pneumoniae* (IgM) | Chronic mucositis | Chronic hepatitis B | Pulmonary infection |
| 64 | *Lautropia mirabilis^a^* | Non | Chronic mucositis | Hypoproteinemia | Pulmonary infection |
| 65 | *Corynebacterium argentoratense^a^*  *Rothia mucilaginosa^a^* | Non | Non | Non | Pulmonary infection |
| 66 | *Neisseria meningitidis* | T-spot  parainfluenza virus (IgM) | Chronic mucositis | Non | Pulmonary infection  Chronic bronchitis  Emphysema |
| 67 | *Stenotrophomonas maltophilia*  *Acinetobacter baumannii*  Human alphaherpesvirus 1^a^  *Aspergillus fischeri* | *Stenotrophomonas maltophilia*  *Acinetobacter baumannii*  *Staphylococcus haemolyticus*  *Haemophilus influenzae* | Non | Acute myocardium infarction (Post- PCI)  Hypertention | Pulmonary infection |
| 68 | *Rothia dentocariosa^a^* | Non | Lung tissue | Membranous nephropathy  Diffuse connective tissue disease | Connective tissue disease associated with interstitial lung disease (CT-ILD) |
| 69 | Non | Non | Chronic mucositis | Aheumatoid arthritis | ICH |
| 70 | *Mycobacterium tuberculosis* | *Tuberculosis* | Mucosal granulomatous inflammation, consider tuberculosis | Ankylosing Spondylitis | ICH  Tuberculosis |
| 71 | *Bordetella pertussis*  *Mycobacterium tuberculosis* | FluA (IgM) | Interstitial mild hyperplasia | Ankylosing Spondylitis | ICH  Tuberculosis? |
| 72 | *Staphylococcus haemolyticus*  Human  alphaherpesvirus 1^a^ | Non | Focal non-small cell lung cancer infiltration | Non | NSCLC (Newly diagnosed) |
| 73 | *Mycobacterium* *tuberculosis* | *Tuberculosis* | Mucosal granulomatous inflammation, consider tuberculosis | Non | Tuberculosis |
| 74 | *Staphylococcus haemolyticus*  Human gammaherpesvirus 4  *Pneumocystis jirovecii* | Non | Organizing pneumonia | Hematopoietic stem cell transplantation | ICH |
| 75 | *Pseudomonas aeruginosa* | *Pseudomonas aeruginosa*  FluB (IgM) | Chronic mucositis | Non | Bronchiectasis with infection |
| 76 | *Streptococcus infantis ^a^*  Human alphaherpesvirus 1 ^a^ | Fungal hypha (sputum smear) | Chronic mucositis | Non | Pulmonary infection  COPD |
| 77 | *Staphylococcus epidermidis ^a^*  *Candida albicans* | Non | Non | Non | Pulmonary infection  COPD |
| 78 | *Pseudomonas aeruginosa*  *Abiotrophia defectiva ^a^* | Non | Non | Non | Pulmonary infection  Idiopathic pulmonary fibrosis  Pulmonary arterial hypertension |
| 79 | Non | *Candida albicans* | Chronic mucositis | Non | Pulmonary abscess |
| 80 | Human betaherpesvirus 5 | FluA (IgM)  CMV | Chronic mucositis | Non | Pulmonary abscess  Asthma |
| 81 | Non | G+ | Mucosal purulent inflammation | Non | Pulmonary abscess |
| 82 | Non | *Tuberculosis* | Chronic mucositis | Non | ICH  NSCLC (after chemotherapy)  Tuberculosis |
| 83 | *Prevotella intermedia ^a^* | Non | Non | Non | Pulmonary infection |
| 84 | *Enterococcus faecium*  Human betaherpesvirus 5  *Pneumocystis jirovecii* | G+ | Non | Kidney transplantation  Coronary heart disease (Post- PCI) | ICH |
| 85 | *Nocardia mikamii*  Human gammaherpesvirus 4 | *Nocardia* | Non | Membranous nephropathy | ICH |
| 86 | *Pseudomonas aeruginosa*  Human betaherpesvirus 5  *Pneumocystis jirovecii* | *Pseudomonas aeruginosa* | Non | IgA nephropathy | ICH  Pulmonary fibrosis |
| 87 | *Mycobacterium tuberculosis*  *Klebsiella pneumoniae* | *Tuberculosis* | Caseous necrosis | Diabetes | Tuberculosis |
| 88 | Non | T-spot+ | Chronic mucositis | Non | Pulmonary infection |
| 89 | *Mycobacterium iranicum* | TB-DNA | Non | Non | Bronchiectasis with hemoptysis  Old tuberculosis |
| 90 | *Mycoplasma pneumoniae* | Non | Chronic mucositis | Non | Pulmonary infection |
| 91 | *Prevotella intermedia ^a^* | Non | Chronic mucositis | Non | Tuberculosis  Chronic bronchitis |
| 92 | *Aspergillus oryzae* | Non | Chronic mucositis | Non | Pulmonary infection  Pulmonary fibrosis |
| 93 | *Streptococcus pneumoniae* | *Streptococcus pneumoniae*  *Candida albicans*  EBV | Non | Multiple myeloma | ICH |
| 94 | *Rothia mucilaginosa ^a^* | *Pseudomonas aeruginosa* | Chronic mucositis | Coronary heart disease | Pulmonary infection |
| 95 | *Pseudomonas aeruginosa*  *Mycoplasma salivarium ^a^*  *Aspergillus oryzae* | Non | Chronic mucositis | Non | Bronchiectasis with infection |
| 96 | *Veillonella parvula ^a^*  Torque teno virus 1  Human betaherpesvirus 5  *Pneumocystis jirovecii* | *Streptococcus hemolyticus* G+  CMV | Non | Kidney transplantation | ICH |
| 97 | Torque teno virus  *Pneumocystis jirovecii* | Non | Non | Kidney transplantation | ICH |
| 98 | *Mycobacterium tuberculosis*  Torque teno virus | *Acinetobacter baumannii*  *Tuberculosis* (Sputum smear) | Non | Non | Tuberculosis |
| 99 | Human alphaherpesvirus 1 ^a^  *Aspergillus niger* | *Candida albicans* | Non | Coronary heart disease (Post- PCI) | Pulmonary infection  Pulmonary fibrosis |
| 100 | *Mycoplasma salivarium ^a^*  *Chlamydia psittaci* | Non | Interstitial fibrous tissue hyperplasia | Non | Pulmonary infection |
| 101 | *Mycobacterium tuberculosis* | *Tuberculosis* | Chronic mucositis | Non | Tuberculosis |
| 102 | *Rothia mucilaginosa ^a^* | Non | Focal granuloma formation. | Acute cerebral infarction  Diabetes | Pulmonary infection |
| 103 | *Klebsiella pneumoniae*  *Mycobacterium tuberculosis*  Human alphaherpesvirus 1 ^a^ | Non | Chronic mucosal inflammation, fibrous exudate | Non | Pulmonary infection  COPD |
| 104 | *Mycoplasma pneumoniae* | Non | Chronic mucositis | Non | Pulmonary infection |
| 105 | *Capnocytophaga sputigena^a^* | Non | Chronic mucositis | Non | Pulmonary infection |
| 106 | *Klebsiella pneumoniae* | Non | Chronic mucositis | Non | Pulmonary infection |
| 107 | *Prevotella melaninogenica ^a^*  *Prevotella pallens ^a^* | Non | Chronic mucositis | Hypertention  Abdominal aortic stenosis | Pulmonary infection |
| 108 | *Mycoplasma pneumoniae* | *Mycoplasma pneumoniae* (IgM) | Chronic mucositis | Non | Pulmonary infection |
| 109 | *Aspergillus niger* | *Aspergillus niger*  Fungus  FluB | Lung tissue | Non | ICH (Chronic eosinophil pneumonia) |
| 110 | *Pneumocystis jirovecii* | FluB | Chronic mucosal inflammation with fibrous tissue hyperplasia | Non | Pulmonary infection  Pleural effusion |
| 111 | *Fusobacterium nucleatum*  *Campylobacter rectus ^a^*  Human gammaherpesvirus 4  Human betaherpesvirus 7 | Non | Non | Non | Pulmonary infection  NSCLC (Newly diagnosed) |
| 112 | *Staphylococcus haemolyticus*  Human gammaherpesvirus 4  *Candida albicans* | *Acinetobacter baumannii*  *Klebsiella pneumoniae* | Non | Cerebral hemorrhage | Pulmonary infection |
| 113 | *Streptococcus pneumoniae*  Human alphaherpesvirus 1 ^a^ | FluB (IgM) | Non | Uremia | Pulmonary infection |
| 114 | Non | *Candida albicans* | Lung tissue | Non | Pulmonary infection |
| 115 | Non | Non | Adenocarcinoma | Coronary heart disease  Hypertention | Pulmonary infection  NSCLC (Newly diagnosed) |
| 116 | *Streptococcus suis*  Human alphaherpesvirus 1 ^a^  *Candida albicans* | *Candida albicans* | Chronic mucosal inflammation with local superficial necrosis | Anemia | Pulmonary infection |
| 117 | Non | *Tuberculosis* | Mucosal granulomatous inflammation, consider tuberculosis | Non | Tuberculosis |
| 118 | *Gemella haemolysans* | Non | Chronic mucositis | Non | Pulmonary infection |
| 119 | *Streptococcus oralis ^a^* | Non | Chronic mucositis | Hypertention  Drug-induced liver injury | Pulmonary infection |
| 120 | Non | Non | A little small cell carcinoma | Coronary heart disease (Post- PCI) | SCLC  COPD |
| 121 | Non | *Stenotrophomonas maltophilia*  *Candida glabrata*  Fungus | Non | Diabetes  AF | Pulmonary infection  COPD |
| 122 | *Weissella confusa ^a^* Human gammaherpesvirus 4 *Aspergillus oryzae* | EBV | Non | Nephrotic syndrome | ICH |
| 123 | *Prevotella pallens ^a^*  *Prevotella melaninogenica ^a^* | *Chlamydia pneumoniae* (IgM) | Chronic mucositis | Non | Pulmonary infection  COPD |
| 124 | *Actinomyces oris ^a^*  *Actinomyces naeslundii ^a^*  Torque teno virus 12  *Candida albicans* | Non | Interstitial fibrous tissue hyperplasia | Hypertention  Diabetes | Pulmonary infection |
| 125 | *Acinetobacter baumannii* | Non | Non | Hypertention | Pulmonary infection |
| 126 | *Mycobacterium tuberculosis* | *Tuberculosis* | Chronic mucosal inflammation with superficial local necrosis and fibrous tissue hyperplasia | Non | Tuberculosis  COPD |
| 127 | *Prevotella pallens ^a^* | Non | Chronic mucositis | Non | Pulmonary infection  COPD |
| 128 | *Campylobacter rectus ^a^* | Non | Fibrous tissue hyperplasia | Non | Pulmonary abscess  Lung cancer? |
| 129 | *Mycoplasma pneumoniae* | Non | Chronic inflammation of lung tissue with fibrous exudation in alveolar cavity | Non | Pulmonary infection |
| 130 | Non | Non | Chronic mucositis | Non | Pulmonary infection  Asthma |
| 131 | Non | Non | Non | Rheumatoid arthritis | ICH  Pulmonary fibrosis |
| 132 | *Prevotella melaninogenica ^a^* | Non | Chronic mucositis | Valvulopathy | Pulmonary infection |
| 133 | Non | Non | Pulmonary interstitial inflammation | Non | Lung cancer (Newly diagnosed)  COPD |
| 134 | *Corynebacterium accolens* | Non | Chronic mucositis | Non | Pulmonary infection  Asthma |
| 135 | *Neisseria meningitidis*  *Actinomyces graevenitzii ^a^* | Non | Squamous cell carcinoma | Non | NSCLC (Newly diagnosed) |
| 136 | Non | Non | Chronic mucositis | Non | Pulmonary infection |
| 137 | Non | Non | Adenocarcinoma | Non | NSCLC (Newly diagnosed) |
| 138 | *Pseudomonas aeruginosa* | Non | Chronic mucositis | Non | Pulmonary nodules |
| 139 | *Mycolicibacillus koreensis*  Human betaherpesvirus 6A ^a^ | G+ | Chronic mucositis | Hypertension | Lung abscess |
| 140 | *Prevotella nigrescens ^a^* | Non | Chronic mucositis | Allergic vasculitis | ICH |
| 141 | *Mycobacterium tuberculosis* | *Tuberculosis* | Mucosal granulomatous inflammation with necrosis, consider tuberculosis | Non | Tuberculosis |
| 142 | *Actinomyces odontolyticus ^a^* | Non | Non | Others | Pulmonary infection |
| 143 | *Enterococcus faecium*  Human alphaherpesvirus 1 ^a^  *Candida parapsilosis* | G+  *Candida parapsilosis*  *Candida krusei* | Non | Non | Pulmonary infection |
| 144 | *Prevotella jejuni ^a^* | G+ | Chronic mucositis | Non | Pulmonary infection |
| 145 | *Prevotella intermedia ^a^*  *Botrytis cinerea* | Non | Chronic mucositis | Non | Pulmonary infection |
| 146 | *Actinomyces odontolyticus ^a^* | Non | Chronic mucositis | Non | Pulmonary infection |
| 147 | *Acinetobacter baumannii*  *Aspergillus flavus* | *Acinetobacter baumannii*  *Aspergillus flavus*  G- | Non | Hypertension | Pulmonary infection |
| 148 | Human alphaherpesvirus 1 ^a^ | *Tuberculosis* | Mucosal granulomatous inflammation with necrosis, consider tuberculosis | Non | Tuberculosis |
| 149 | *Mycobacterium tuberculosis* | *Tuberculosis* | Mucosal granulomatous inflammation with necrosis, consider tuberculosis | Non | Tuberculosis |
| 150 | *Rothia dentocariosa ^a^* | Non | Chronic mucositis | Non | Bronchiectasis with infection  Old tuberculosis |
| 151 | *Haemophilus influenzae*  *Moraxella catarrhalis* | Non | Chronic mucositis | Non | Pulmonary infection |
| 152 | *Porphyromonas*  *gingivalis ^a^* | G+  G-  *Mycoplasma* (IgM) | Chronic mucositis | Diabetes | Pulmonary infection |
| 153 | *Mycobacterium tuberculosis* | *Tuberculosis* | Mucosal granulomatous inflammation with necrosis, consider tuberculosis | Non | Tuberculosis |
| 154 | *Fusobacterium periodonticum ^a^*  Human mastadenovirus B | FluA(IgM) | Chronic mucositis | Others | Pulmonary infection |
| 155 | *Pseudomonas aeruginosa* | *Aspergillus flavus*  *Candida albicans* | Chronic mucositis | Non | Pulmonary infection |
| 156 | *Enterococcus faecium Pseudomonas aeruginosa* | *Pseudomonas aeruginosa* | Non | Inflammatory myopathy | ICH |
| 157 | *Prevotella intermedia ^a^* | Non | Chronic mucositis | Non | Pulmonary infection |
| 158 | *Pseudomonas aeruginosa* | Non | Chronic mucositis | Non | Lung cancer? |
| 159 | *Prevotella melaninogenica ^a^*  *Porphyromonas endodontalis ^a^* | Non | Non | Others | Pulmonary infection |
| 160 | *Mycobacterium tuberculosis* | *Tuberculosis* | Mucosal granulomatous inflammation with necrosis, consider tuberculosis | Non | Tuberculosis |
| 161 | *Haemophilus influenzae* | Non | Chronic mucositis | Non | Pulmonary infection |
| 162 | *Prevotella jejuni ^a^*  Human alphaherpesvirus 1 ^a^ | *Aspergillus fumigatus* | Non | Non | Pulmonary infection  COPD |
| 163 | *Prevotella jejuni ^a^* | Non | A little lung tissue | Non | Pulmonary infection |
| 164 | Non | *Tuberculosis* | Non | Hypertension  Diabetes | Tuberculosis |
| 165 | *Staphylococcus aureus* | FluA (IgM) | Chronic mucositis | Non | Pulmonary infection  COPD |
| 166 | *Prevotella intermedia ^a^* | Non | Non | Non | ICH  NSCLC (after chemotherapy) |
| 167 | *Actinomyces oris ^a^* | Non | Adenocarcinoma | Diabetes  Coronary heart disease | NSCLC (Newly diagnosed) |
| 168 | *Klebsiella pneumoniae* | *Tuberculosis* | Chronic mucositis | Non | Tuberculosis |
| 169 | *Stenotrophomonas*  *maltophilia* | Non | Adenocarcinoma | Non | NSCLC (Newly diagnosed) |
| 170 | *Acinetobacter*  *baumannii* | Non | Chronic mucositis | Non | Pulmonary infection |
| 171 | Non | Non | Chronic mucositis | Non | Bronchiectasia  Old tuberculosis |
| 172 | *Staphylococcus aureus* | *Mycoplasma pneumoniae* (IgM)  Adenovirus (IgM) | Chronic mucositis | Systemic lupus erythematosus | ICH |
| 173 | *Staphylococcus aureus*  *Pseudomonas aeruginosa* | G+ | Chronic mucositis | Coronary heart disease | Pulmonary infection  Asthma |
| 174 | *Actinomyces odontolyticus ^a^* | *Mycoplasma pneumoniae* (IgM) | Chronic mucositis | Non | Pulmonary infection  NSCLC（Post pneumonectomies） |
| 175 | *Stenotrophomonas maltophilia*  Human alphaherpesvirus 1 ^a^ | *Stenotrophomonas maltophilia* | Non | Non | Pulmonary infection |
| 176 | *Streptococcus pneumoniae* | Non | Non | Non | Pulmonary infection |
| 177 | *Mycolicibacterium tusciae* | G+ | Non | Sicca syndrome | ICH |
| 178 | *Streptococcus pneumoniae* | *Streptococcus pneumoniae* | Non | Agranulemia | ICH |
| 179 | *Acinetobacter baumannii* | G+ | Non | Non | Pulmonary infection |
| 180 | *Staphylococcus aureus*  *Corynebacterium striatum*  *Candida albicans* | G+  FluA | Chronic mucositis | Coronary heart disease | Pulmonary infection |
| 181 | *Fusobacterium nucleatum* | Non | Chronic mucositis | Non | Bronchiectasis and infection  COPD |
| 182 | *Neisseria mucosa ^a^*  Human gammaherpesvirus 4  *Pneumocystis jirovecii* | Non | Non | Kidney transplantation | ICH |
| 183 | *Pseudomonas aeruginosa* | Non | Non | Diabetes | Pulmonary infection |
| 184 | *Klebsiella pneumoniae* | Non | Chronic mucositis | Non | Pulmonary infection |
| 185 | *Aspergillus flavus*  *Aspergillus fumigatus* | *Aspergillus flavus*  *Candida albicans* | Non | Non | Pulmonary infection  COPD  Asthma |
| 186 | *Rothia mucilaginosa ^a^*  *Streptococcus oralis ^a^* | FluB | Adenocarcinoma | Non | Pulmonary infection  NSCLC (Newly diagnosed) |
| 187 | *Streptococcus intermedius ^a^* | *Candida albicans* | SCLC | Non | Pulmonary infection  SCLC (Newly diagnosed) |
| 188 | *Streptococcus pneumoniae*  Human gammaherpesvirus 4 | Non | Chronic mucositis | Non | Pulmonary infection |
| 189 | *Fusobacterium periodonticum ^a^* | Non | Chronic mucositis | Others | Pulmonary infection |
| 190 | *Chlamydia psittaci* | *Mycoplasma pneumoniae* (IgM) | Chronic mucositis | Others | Pulmonary infection |
| 191 | *Actinomyces graevenitzii ^a^*  *Mycoplasma salivarium* | *Tuberculosis* | Mucosal granulomatous inflammation with necrosis, consider tuberculosis | Diabetes  Hypertension | Tuberculosis |
| 192 | Non | Non | Non | Non | Pulmonary infection  COPD |
| 193 | *Pseudomonas aeruginosa*  *Cyberlindnera jadinii ^a^* | Non | Chronic mucositis | Hypertension | Pulmonary infection |
| 194 | *Fusobacterium nucleatum* | Non | Chronic mucositis | Coronary heart disease | Pulmonary infection |
| 195 | *Pseudomonas aeruginosa*  *Acinetobacter baumannii*  *Aspergillus fumigatus* | Non | Non | Vasculitis | ICH |
| 196 | *Actinomyces odontolyticus ^a^* | Non | Non | Hypertension | Pulmonary infection  COPD |
| 197 | *Aspergillus fumigatus* | *Aspergillus fumigatus*  FluA | Non | Others | Pulmonary infection  COPD |
| 198 | *Enterococcus faecium ^a^*  Human alphaherpesvirus 1 ^a^ | Non | Adenocarcinoma | Hypertension | Pulmonary infection  NSCLC (Newly diagnosed) |
| 199 | *Chlamydia pneumoniae* | *Chlamydia pneumoniae* (IgM) | Chronic mucositis | Others | Pulmonary infection |
| 200 | *Haemophilus influenzae* | Non | Non | Others | Pulmonary infection |
| 201 | Non | Non | Chronic mucositis | Rheumatoid arthritis | ICH |
| 202 | Non | Non | Squamous carcinoma | Non | NSCLC (Newly diagnosed) |
| 203 | Non | FluA | Chronic mucositis | Cerebrovascular disease | Pulmonary infection |
| 204 | *Streptococcus pneumoniae* | *Tuberculosis* | Mucosal granulomatous inflammation with necrosis, consider tuberculosis | Non | Tuberculosis |
| 205 | *Mycobacterium tuberculosis* | *Tuberculosis*  FluB | Mucosal granulomatous inflammation with necrosis, consider tuberculosis | Kidney transplantation | ICH Tuberculosis |
| 206 | *Actinomyces graevenitzii ^a^* | Non | Non | Others | Pulmonary infection |
| 207 | *Mycobacterium tuberculosis*  *Prevotella intermedia ^a^* | *Tuberculosis* | Chronic mucositis | Cerebrovascular disease | Tuberculosis |
| 208 | *Pseudomonas aeruginosa* | *Candida albicans* | Non | Rheumatoid arthritis | ICH |
| 209 | *Bacteroides heparinolyticus ^a^*  *Parvimonas micra ^a^* | Non | Chronic mucositis | Others | Pulmonary infection |
| 210 | *Mycobacterium tuberculosis* | *Tuberculosis*?  EBV | Non | Rheumatoid arthritis  Sicca syndrome  Chronic kidney diseases | ICH  Tuberculosis? |
| 211 | *Enterococcus faecium ^a^*  *Aspergillus fumigatus* | G+  Fungus | Non | Non | Pulmonary infection  Asthma |
| 212 | *Aspergillus fumigatus* | G-  G+ | Fibrous exudate in alveolar cavity | Hypertension | Pulmonary infection |
| 213 | *Pseudomonas aeruginosa* | *Pseudomonas aeruginosa* | Lung tissue necrosis and inflammatory exudation show bacterial flora | Non | Pulmonary infection  NSCLC（Post pneumonectomies） |
| 214 | *Enterococcus faecium ^a^*  *Corynebacterium striatum*  Human gammaherpesvirus 4  Human alphaherpesvirus 1 ^a^  *Candida albicans* | *Corynebacterium striatum*  *Acinetobacter baumannii*  Fungus | Non | Others | Pulmonary infection |
| 215 | *Actinomyces odontolyticus ^a^* | Non | Adenocarcinoma | Non | NSCLC (Newly diagnosed) |
| 216 | *Streptococcus salivarius* | Non | Adenocarcinoma | Hypertension  Others | Pulmonary infection  NSCLC (Newly diagnosed) |
| 217 | *Klebsiella pneumoniae*  *Candida tropicalis*  *Candida glabrata* | *Acinetobacter baumannii*  Fungus | Non | Coronary heart disease  Others | Pulmonary infection |
| 218 | Human betaherpesvirus 5  *Pneumocystis jirovecii* | Fungus | Non | Idiopathic thrombocytopenic purpura | ICH |
| 219 | *Streptococcus pneumoniae*  *Corynebacterium propinquum ^a^* | Non | Non | Others | Pulmonary infection |
| 220 | *Streptococcus pneumoniae* | *Mycoplasma pneumoniae* (IgM) | Fibrous exudation in the alveolar cavity, suggesting infection | Diabetes  Others | Pulmonary infection |
| 221 | *Moraxella catarrhalis* | Non | Adenocarcinoma | Non | NSCLC (Newly diagnosed) |
| 222 | *Haemophilus influenzae*  Human gammaherpesvirus 4 | FluA | Local fibrous tissue hyperplasia | Others | Pulmonary infection |
| 223 | *Pneumocystis jirovecii* | Fungus | Non | Dermatomyositis  Diabetes  Others | ICH |
| 224 | *Acinetobacter baumannii*  *Enterococcus faecalis ^a^* | *Acinetobacter baumannii*  *Klebsiella pneumoniae* G+ | Chronic mucositis | Hypertension  Cerebrovascular disease  Others | Pulmonary infection  Asthma |
| 225 | *Enterococcus faecium* | G+ | Non | Lung transplantation Hypertension  Others | ICH |
| 226 | *Veillonella parvula ^a^* | Non | Chronic mucositis | Coronary heart disease  Cerebrovascular disease | Pulmonary infection |
| 227 | *Corynebacterium striatum*  *Staphylococcus aureus* | *Corynebacterium striatum*  G+ | Non | Non | Pulmonary infection |
| 228 | *Stenotrophomonas maltophilia* | *Acinetobacter baumannii* | Non | Others | Pulmonary infection |
| 229 | *Mycobacterium tuberculosis* | *Tuberculosis* (Sputum smear) | Non | Diabetes | Tuberculosis |
| 230 | *Actinomyces gerencseriae ^a^*  *Actinomyces naeslundii ^a^* | Non | Adenocarcinoma | Coronary heart disease  Cerebrovascular disease | NSCLC (Newly diagnosed) |
| 231 | *Streptococcus pneumoniae* | G+ | Non | Coronary heart disease  Others | Pulmonary infection  Asthma |
| 232 | Non | *Chlamydia pneumoniae* (IgM) | Non | Non | Pulmonary infection |
| 233 | *Enterococcus faecalis ^a^* | Non | Non | Others | Pulmonary infection |
| 234 | Non | Non | Chronic mucositis | ANCA associated systemtc Vasculitis | ICH |
| 235 | *Enterococcus faecium*  *Mycolicibacterium obuense ^a^*  *Pseudomonas aeruginosa*  *Pneumocystis jirovecii*  *Aspergillus flavus* | Fungus | Non | Kidney transplantation | ICH |

^a^ Rare pathogen which was not interpreted as pathogenic microbes.
