# Supplementary material for: Application of Metagenomic Next-Generation Sequencing in the Diagnosis of Pulmonary Infectious Pathogens From Bronchoalveolar Lavage Samples
Source: Front Cell Infect Microbiol. 2021 Mar 11;11:541092. doi: 10.3389/fcimb.2021.541092 (PMC7991794; doi:10.3389/fcimb.2021.541092)
Supplement: Supplementary file 2 [file Table_2.docx]

Supplemental Table S2: The detail information of samples with conflicting results.

| ID | mNGS positive pathogen | Smear results | Culture results | Pathology results | Other laboratory-based diagnostic testing results | Pulmonary disorders |
| --- | --- | --- | --- | --- | --- | --- |
| 8 | Torque teno virus  *Pneumocystis jirovecii* | Negative | *Candida albicans* | Non | Negative | ICH |
| 10 | *Rothia mucilaginosa^a^* | Negative | *Stenotrophomonas maltophilia* | Chronic mucositis | Negative | Pulmonary infection  Pleural effusion |
| 13 | *Actinomyces oris^a^* | Negative | *Candida albicans* | Chronic mucositis | Negative | Fungal infection |
| 17 | *Actinomyces odontolyticus^a^*  Human gammaherpesvirus 4  Human betaherpesvirus 5  *Pneumocystis jirovecii* | Negative | *Acinetobacter baumannii* | Non | Negative | ICH |
| 30 | *Enterococcus faecium^a^* | Negative | *Aspergilus flavus* | Non | Negative | Pulmonary infection |
| 47 | *Mycobacterium triviale*  Human betaherpesvirus 6A | Negative | *Corynebacterium striatum*  *Candida* | Non | Negative | Pulmonary infection |
| 50 | *Rothia mucilaginosa^a^* | Negative | *Acinetobacter baumannii* | Non | Negative | Pulmonary infection |
| 51 | *Pneumocystis jirovecii* | Negative | *Acinetobacter baumannii* | Non | Negative | ICH |
| 94 | *Rothia mucilaginosa ^a^* | Negative | *Pseudomonas aeruginosa* | Chronic mucositis | Negative | Pulmonary infection |
| 96 | *Veillonella parvula ^a^*  Torque teno virus 1  Human betaherpesvirus 5  *Pneumocystis jirovecii* | Gram-positive coccus | *Streptococcus hemolyticus* | Non | CMV positive by quantitative real-time PCR | ICH |
| 98 | *Mycobacterium tuberculosis*  Torque teno virus | Acid-fast staining | *Acinetobacter baumannii* | Non | Negative | Tuberculosis |
| 99 | Human alphaherpesvirus 1 ^a^  *Aspergillus niger* | Negative | *Candida albicans* | Non | Negative | Pulmonary infection  Pulmonary fibrosis |
| 112 | *Staphylococcus haemolyticus*  Human gammaherpesvirus 4  *Candida albicans* | Negative | *Acinetobacter baumannii*  *Klebsiella pneumoniae* | Non | Negative | Pulmonary infection |
| 155 | *Pseudomonas aeruginosa* | Negative | *Aspergillus flavus*  *Candida albicans* | Chronic mucositis | Negative | Pulmonary infection |
| 162 | *Prevotella jejuni ^a^*  Human alphaherpesvirus 1 ^a^ | Negative | *Aspergillus fumigatus* | Non | Negative | Pulmonary infection  COPD |
| 187 | *Streptococcus intermedius ^a^* | Negative | *Candida albicans* | SCLC | Negative | Pulmonary infection  SCLC (Newly diagnosed) |
| 208 | *Pseudomonas aeruginosa* | Negative | *Candida albicans* | Non | Negative | ICH |
| 217 | *Klebsiella pneumoniae*  *Candida tropicalis*  *Candida glabrata* | Fungal hypha | *Acinetobacter baumannii* | Non | Negative | Pulmonary infection |
| 228 | *Stenotrophomonas maltophilia* | Negative | *Acinetobacter baumannii* | Non | Negative | Pulmonary infection |
